# OXIDATIVE STRESS LEVEL AND TYROSINASE ACTIVITY IN VITILIGO PATIENTS

**DOI:** 10.4103/0019-5154.60344

**Published:** 2010

**Authors:** M Eskandani, J Golchai, N Pirooznia, S Hasannia

**Affiliations:** *The University of Guilan, Rasht, Iran.*; 1 *The Guilan University of Medical Science, Guilan, Rasht, Iran.*

**Keywords:** *Comet assay*, *oxidative stress*, *tyrosinase*, *vitiligo*

## Abstract

**Background:**

Vitiligo is an acquired pigmentary disorder of the skin. Genetic factors, oxidative stress, autoimmunity, and neurochemical agents might be contributing factors for the development of the disease.

**Aims:**

To evaluate the oxidative stress level and tyrosinase activity in vitiligo patients and to compare them with healthy volunteers.

**Materials and Methods:**

We used Comet assay to evaluate DNA strand breaks in peripheral blood cells of active vitiligo patients. We then extracted total protein from lesional and nonlesional skin of ten selected patients. Tyrosinase activity was found to play a crucial role in melanogenesis.

**Results:**

The basal level of systemic oxidative DNA strand breaks in leukocytes increased in vitiligo patients compared to healthy participants. We observed that tyrosinase activity in lesional skin was lower than in nonlesional skin.

**Conclusion:**

Our finding suggests that increased levels of oxidative stress might impact tyrosinase activity and eumelanin synthesis via anabolism pathway of melanin synthesis. In sum, we observed a negative correlation between levels of systemic oxidative stress and of tyrosinase activity.

## Introduction

Vitiligo is an acquired pigmentary skin disorder characterized by circumscribed white spots on the skin surface.[1] The lesions may be progressive and may develop at any age.[2] Absence of melanocytes from lesional skin has been suggested as a cause of white spots.[3] The etiology is still unknown but various hypotheses have been proposed. Some of these are:

1. The genetic hypothesis that emphasizes intrinsic inherent melanocyte defect.[4]
2. The melanogenesis pathway and enzymes defect.
3. Induction of melanogenesis by melanocortin (melanocyte stimulating hormone) and melanocortin-1 receptor (MC1R) defect.
4. cAMP signaling pathway defect.

The melanogenic actions of melanocortins are mediated by the MC1R.[5] MC1R is a member of the G-protein-coupled receptors (GPCRs) super family expressed in cutaneous and hair follicle melanocytes. Activation of MC1R by adrenocorticotropin or α-melanocyte stimulating hormone (α-MSH) that results from cleavage of Pro-opiomelanocortin (POMC) is positively coupled to cAMP signaling pathway. This activation leads to stimulation of melanogenesis and disruption of pheomelanin synthesis resulting in the production of eumelanic pigments. These pigment cells which have tyrosinase activity are key Cells in melanogenesis.[6] Tyrosinase is one of the important enzymes that has a key role in pigmentation process.[7] Tyrosinase is a sensitive enzyme and, as a result, a range of factors can influence its activity. In spite of its sensitivity, investigators have shown that the tyrosinase of mushrooms can use oxidative agents such as H_2_ O_2_ as a secondary substrate.[8] The oxidative stress hypothesis suggests that melanocyte impairment could be related to an increased oxidative stress with consequent induction of H_2_ O_2_ accumulation in the epidermis of active vitiligo patients.[9] Defective recycling of tetrahydrobiopterin (BH_4_) in vitiligo epidermis is associated with the intracellular production of H_2_ O_2_.[10] Lower levels of antioxidant systems such as catalase or vitamin A, C, and E were demonstrated in the epidermis of both lesional and nonlesional skin of vitiligo patients.[11] An increased intracellular production of reactive oxygen species (ROS) that can interact with macromolecules such as proteins, membrane lipids, and nucleic acids due to mitochondrial impairment was observed in diseased patients.[12]

The study aimed to evaluate the oxidative stress level and tyrosinase activity in vitiligo patients and to compare them with healthy volunteers. In this study, the extent of DNA damage was assessed by Comet assay and the percentage of DNA in the tail region is linearly related to the damaged DNA which in turn reflects higher systemic oxidative stress. A comparison of the tyrosinase activity data and Comet assay data will elaborate a probable correlation between oxidative stress and tyrosinase activity.

## Materials and Methods

### Participants

Participants comprised 21 active nonsegmental vitiligo patients and 21 healthy controls. Comet assay was performed on blood samples from age and sex matched participants. There were 12 men and nine women in each group, their ages ranging from 15-40 years.

### Blood collection

Peripheral blood samples (total 1.5 ml) were obtained from all the participants. The blood samples were stored in EDTA-containing eppendorf at 10°C and kept in a dark room to prevent further DNA damage. For analysis of DNA damage in leucocytes, 5 μl of fresh whole blood was transferred to eppendorf tube, mixed with 75 μl of 37°C low melting point agarose, and layered into precoated slide with normal melting point agarose. After allowing the agarose to solidify, the slides were subjected to Comet assay.[13]

### Comet assay

The slides with the agarose-embedded cells were subjected to a lysis step (4 h incubation at 4°C in 2.5 M NaCl, 100 mM Na_2_ EDTA, 1% triton X-100, and 10% DMSO (pH 10.5)). The slides were then placed in an ice-cold electrophoresis chamber containing alkaline electrophoresis solution (300 mM NaOH, 1 mM Na_2_ EDTA of pH > 13) for 40 min to allow DNA to unwind. This was followed by electrophoresis conducted for 20 min at 300 mA and 20 V. At the end of electrophoresis, the slides were washed with neutralization buffer (40 mM Tris-HCl, pH 7.5), stained with a drop of ethidium bromide, and covered with 20 × 20 cover slip for an immediate microscopic analysis.

Microscopic analysis was carried out by means of an Olympus microscope (Bh2-RFCA, Japan) provided with epifluorescence (wavelength 546 nm; barrier 580 nm). The image of 100 randomly chosen nuclei for every two slides were captured and analyzed with CASP software. For each image, the program calculated the total fluorescence distribution of head and tail of the comet, respectively. DNA strand breaks were expressed as the percentage of total fluorescence migrated in the tail for each nucleus (% DNA in tail/% DNA in head).

### Dose response for H_2_ O_2_

In order to evaluate the response of leukocytes to a DNA-damaging agent and measuring of systemic antioxidant properties, whole blood slides obtained from four vitiligo patients and four controls were exposed to H_2_ O_2_ (from 0-100 mM in PBS). As a reference, slides of both patients and controls were incubated in PBS. The incubation with H_2_ O_2_ was conducted for 15 min, at 4°C to inhibit DNA repair. The slides were then coated with agarose and once the gel solidified they were immediately immersed in a large volume (100 ml per slide) of PBS containing H_2_ O_2_ at the desired concentration. After completing the incubation with H_2_ O_2_, the slides were transferred to the lysis solution and run through the rest of the procedure as described above.

### Participants for tyrosinase assay

Ten active nonsegmental vitiligo patients were selected and a lesional and nonlesional skin sample was obtained from each of them. The obtained sample from each participant was weighed about 40 mg. Each skin sample was minced thoroughly with a pair of scissors and homogenized at 1-min intervals for 3 min in 1.3 ml of 0.02 M sodium pyrophosphate, pH 7.4, with a microtip sonicator (heat systems, plainview, NY) and intensity of 10 chilled in ice (Ivan Sorvall Inc., Norwalk, Conn.). Homogenates were then left at 4°C for 1 h prior to centrifugation. In order to determine the cellular distribution of tyrosinase and the effect of detergents on enzyme volatilization, homogenates were centrifuged at 20000 g for 40 min at 4°C. The supernatants were removed for assay and the pellets resuspended by sonication in phosphate buffer prior to assay.[14]

### Dialysis

The supernatants containing tyrosinase were dialyzed against 1 liter of 0.02 M sodium phosphate buffer as previously described,[15] pH 6.8, for 12 h, with four changes of buffer after evert 3 h at 4°C.

### Electrophoretic and gel specific activity staining procedures

Analytical SDS/PAGE was performed as described in,[16] in 12% acrylamide gels, but without 2-mercaptoethanol and heating to preserve tyrosinase activity. Samples were mixed in a 2:1 ratio with sample buffer (0.18 M Tris/ HCl, pH 6.8/15% glycerol/0.075% Bromophenol Blue/9% SDS), and electrophoresis was carried out at 4°C. A highly sensitive and specific diphenol oxidase activity stain was carried out by equilibrating the gels at pH 6.0 with 50 mM sodium phosphate buffer, followed by incubation at 37°C in 1.5 mM l-dopa/4 mM MBTH, in 10 mM phosphate buffer, pH 6.8, from 15-30 min.[16]

### Measurement of diphenol oxidase activity of tyrosinase

#### Preparation of substrate solution

Dopamine hydrochloride (44 mM) was freshly prepared in phosphate buffer (pH 6.8) containing 2% (v/v) DMF and 5 mM MBTH to prevent its color change by the action of direct light. This solution was stored in dark until use.

### Protein determination

Protein concentration was determined by the method of Bradford and *et al*.[17]

### Enzyme assay

The enzymatic reaction was initiated by addition of known amount of the skin extract to a solution of substrate containing dimethyl formamide DMF and MBTH. DMF was included in the reaction mixture in order to keep the resulting colored complex in solution state during the course of our investigations. The progress of the reaction was followed by measuring the intensity of the resulting pink color at 503 nm. A typical reaction mixture was generated with total volume of 1.0 ml contained 60 μl cell extract, 500 μl substrate solution, and 440 μl phosphate buffer (pH 6.8).[18]

## Results

### Comet assay

**Figure 1:**
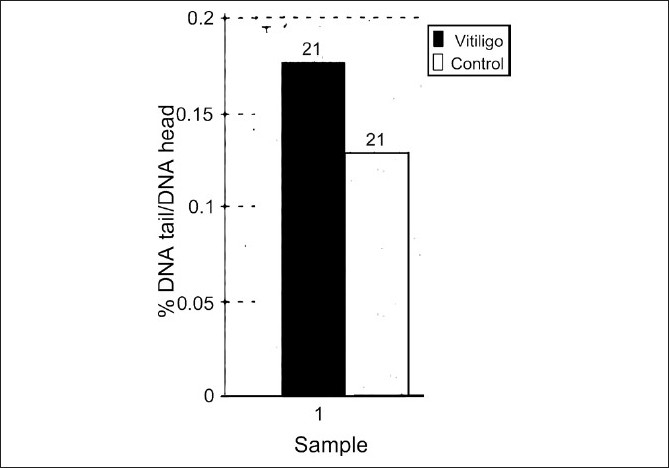
DNA damage in nonisolated leukocytes, analyzed in whole blood slides prepared from vitiligo patients (black columns) and controls (white columns). DNA damage is expressed as the mean ± S.E. (*n* = 21) of the percent DNA migrated in the tail of the comet (%DNA in tail) to head of comet. The basal levels of DNA damage (breaks) are shown. *P < 0.05 statistically significant difference between vitiligo and controls (Student's *t-test*)

Comet assay was carried out on all the participants and as shown in Figure 1, the oxidative breaks of DNA strands in patients was high compared to control participants (*P* < 0.05).

**Figure 2:**
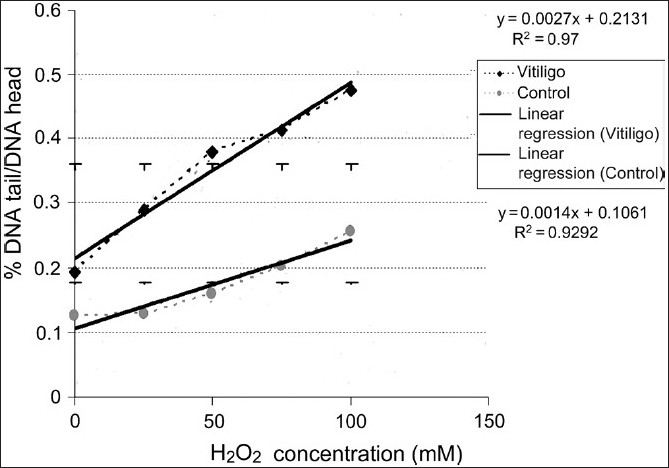
*In vitro* induced DNA damage in nonisolated leukocytes, analyzed in whole blood slides prepared from vitiligo patients (black dots) and controls (gray dots), exposed to increasing concentrations of H_2_O_2_ (from 1-100 mM)

A dose-dependent increase in DNA damage was found upon H_2_ O_2_ exposure (slopes significantly different from zero for both curves, *P* < 0.05). Linear regression analysis also showed that the slopes of the two curves were statistically different from each other, indicating that the response to *in vitro* oxidative stress was not similar in vitiligo patients and control subjects. The findings of this study revealed that patients had reduced systemic antioxidant defense owing to increased levels of oxidative agents resulting in undesired response by the *in vitro* live leukocytes compared to controls subjects [Figure 2].

### Gel specific staining and direct spectrophotometric assay of diphenol oxidase activity of tyrosinase

**Figure 3:**
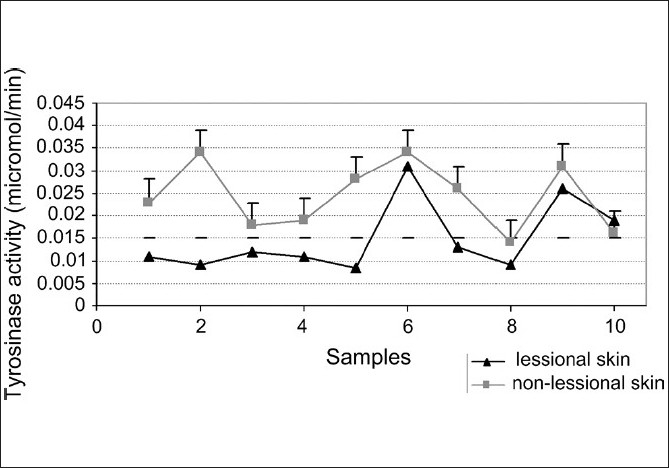
Specific activity assay of tyrosinase showed that activity of lesional tyrosinase was lower than nonlesional tyrosinase

**Figure 4:**
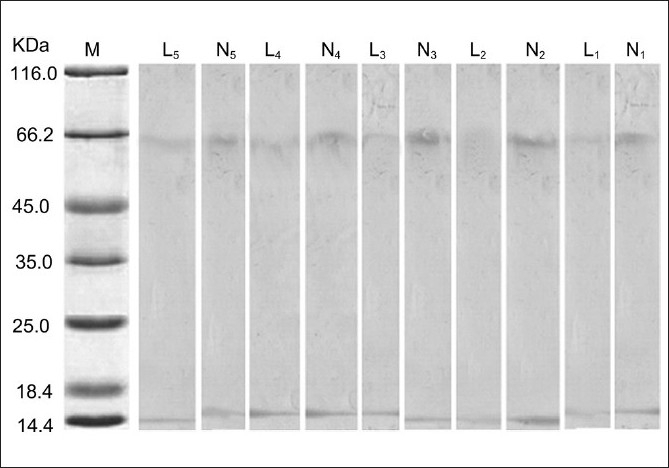
Specific gel staining of tyrosinase in five nonlesional and lesional skins of vitiligo patients. N - the total protein in nonlesional skins, L - The total protein in lesional skins, M - Protein molecular marker

Assay of specific diphenoloxidase activity of cell extract showed that the basal activity of tyrosinase in lesional skins of nonsegmental vitiligo patients were lower than nonlesional skins [Figure 3]. Gel specific staining of tyrosinase was performed and we observed that pink color for specific stain in nonlesional skins in five patients was higher than in their lesional skins [Figure 4].

## Discussion

An important determinant of skin cancer risk is cutaneous pigmentation and the ability to tan upon sun exposure. In active vitiligo patients an increased oxidative stress of the entire epidermal compartment has been demonstrated.[19] In particular, catalase activity, reduced glutathione, and Vitamin E levels decreased[20] and this imbalance of antioxidants was associated with hyperproduction of ROS.[21] For the first time, in 2004 a report concerning the assessment of oxidative DNA damage in vitiligo patients was published, showing of the quantity of DNA breakage in mononuclear leukocytes of vitiligo patients being comparatively higher.[9]

In our study, the results of indirect Comet assay for the level of damaged DNA and hence systemic oxidative stress showed higher amounts of stress in vitiligo patients compared to healthy participants. Generally, oxidative stress in cells is caused due to:

1. The reduced function or the defective antioxidative system of the body and
2. The presence of ROS which is produced during biochemical reactions in the body.

Studies have shown that the skin of vitiligo patients can contain high amounts of ROS which are produced as a result of mitochondrial defects.

In the current study, analysis of the action of oxidative agents such as H_2_ O_2_ in varying doses on whole blood obtained from patients and healthy individuals revealed that immune antioxidant systems of patients were comparatively reduced [Figure 2].

These findings reveal that probably the reduced functionality and oxidative defect in Iranian patients is one of the reasons for development of vitiligo in these individuals. Therefore, probably the treatment of these individuals with antioxidants can help prevent or cure the condition.

Also, one of the important enzymes that has a key role in pigmentation process is tyrosinase. Tyrosinase is a sensitive enzyme that a range of materials can influence on its activity. We assayed tyrosinase activity in ten lesional and nonlesional skins of vitiligo patients and observed that tyrosinase activity in lesional skins of patients was lower than nonlesional skins [Figure 2].

In spite of recent research revealing that the tyrosinase enzyme obtained from mushrooms can use oxidative agents such as H_2_ O_2_ substrate, the current study shows that the amount of tyrosinase activity present in the total protein of patients with healthy and vitiligo affected skin becomes significantly affected in the presence of H_2_ O_2_.

The explanation provided for this phenomenon is that H_2_ O_2_ in the presence of Dopa substrate and MBTH can function as tyrosinase enzyme inhibitor or the presence of H_2_ O_2_ only and Dopa substrate can generate secondary complex which can bind and inhibit the enzyme. One of the other reasons of this phenomenon can be attributed to the different isozymes of tyrosinase and the ability of each to absorb one substrate.

When tyrosinase data and Comet assay data were compared there seemed to exist a probable relation between the oxidative stress levels and tyrosinase activity as the level of oxidative stress was higher than normal in vitiligo patients and the tyrosinase activity in their lesional skins was lower than normal. Therefore, a clinical study that confirms this data can be useful for vitiligo studies and its treatments.

## Conclusion

This study supports the hypothesis that in vitiligo patients a systemic oxidative stress can exist. The studies revealed that an increase in DNA damage in leucocytes of patients and that a dose response of leucocytes to oxidative agent was lower than control subjects. This data support the hypothesis that one of the reasons of oxidative damage in vitiligo patients can be lower defense antioxidant system such as Vitamin E and so on. Also, this study showed that tyrosinase activity in lesional skins of vitiligo patients was lower than their nonlesional skins and a meaningful correlation can be exist between increased of oxidative stress and decreased tyrosinase activity. Probably in the reasoning of this phenomenon we can say that there exists a role of oxidative agent in the inhibition of tyrosinase activity.
